# Navigating the broken road: A call to strengthen access, equity, and inclusivity in the care of children with developmental disabilities and neurobehavioral disorders

**DOI:** 10.34172/hpp.2022.44

**Published:** 2022-12-31

**Authors:** Aysha Jawed, Christine Peck

**Affiliations:** Johns Hopkins Children’s Center, Department of Pediatric and OB/GYN Social Work, Baltimore, MD, USA

**Keywords:** Neurobehavioral disorder, Developmental disability, Pediatric, Health equity accessibility, Inclusivity, Diversity, Healthcare

## Abstract

There is a significant scarcity of resources to achieve behavioral stabilization among children and adolescents with moderate to severe developmental disabilities and neurobehavioral disorders. In total, there are currently 76 inpatient pediatric neurobehavioral programs to support these patients across the United States. Many states do not currently have programs of this nature. Across existing programs, there are substantial waiting lists. In addition, non-public school, intensive day program, in-home and additional outpatient services are not reaching these patients fast enough which further exacerbate the sequalae of suboptimal outcomes and future quality of life implications for these patients. In addition, disparities remain in how the chronicity of developmental disabilities and neurobehavioral disorders are addressed within our healthcare system. It is crucial to categorize this constellation of specialized conditions as chronic illnesses which warrant continued care and treatment, similar in nature to lifelong medical conditions. Further time and priority are warranted in increasing accessibility, equity, and inclusivity in our U.S. healthcare system to optimize a range of health and developmental outcomes for these patients. Future work in this domain could also contribute towards the larger goal of the World Health Organization, Healthy People 2030, and the Sustainable Development Goals of the United Nations in securing delivery of healthcare services that are inclusive, equitable and accessible for individuals with disabilities.

## Introduction

###  Descriptive epidemiology and classifications 

 A developmental disability represents a condition or disorder attributed to an impairment in physical, learning, language, and/or behavior and can range in severity on a spectrum.^1^ Furthermore, a developmental disability can result in significant global developmental delays in meeting milestones and cognitive functioning as well as impact activities of daily living. The trajectory of a developmental disability is chronic and lifelong in nature. Autism or autism spectrum disorder is a prevalent developmental disability that accounts for the range of conditions characterized by challenges in social skills, repetitive behaviors, speech and nonverbal communication.^2^ 1 in 6 children between 3 to 17 years of age have one or more developmental disabilities in the United States.^1^ In addition, 1 in 44 children are affected by Autism based on estimates from the CDC’s Autism and Developmental Disabilities Monitoring (ADDM) Network.^2^

###  Health inequities in existing subacute resources 

 There are several national foundations (e.g. March of Dimes, Autism Speaks, The Arc) that engage in research and advocacy endeavors. However, there are regional limitations in reaching segments of this population who could benefit from these resources the most given the gaps in outreach. Currently across the country, the most widely visible community programs involve in-home services, intensive day programs, specialized schools, and inpatient neurobehavioral programs.

 Since the inception of the Developmental Disabilities Act and subsequent legislation that followed thereafter, community health policies present opportunities for patients with developmental disabilities and neurobehavioral disorders to be eligible for diverse waiver programs that will waive financial eligibility criteria to optimize access to resources and services.^3,4^ In addition based on the Rehabilitation Act of 1973, every school district is required to provide free appropriate public education to all students with disabilities, irrespective of the nature or severity of a student’s disability. This federal legislation extends to jurisdictions across all states. However, the limiting factors in the implementation of these policies are the significant resource limitations and waiting lists across community programs.

 We live in a world where our healthcare system is not built for these patients. Health inequities directly stem from significant limitations in access to care. There is a significant scarcity of subacute (inpatient) behavioral stabilization programs across the country which further contributes towards exacerbating the sequalae of suboptimal health, psychological, socioemotional, developmental, and academic outcomes among children with moderate to severe developmental disabilities and neurobehavioral disorders. We delineate the existing disparities and fragmentation in the delivery of inpatient and outpatient services for these children.

## Inpatient pediatric neurobehavioral programs

 These subacute behavioral stabilization programs comprise intensive inpatient neurobehavioral units for children with severe developmental disabilities and neurobehavioral disorders within a healthcare institution. The overarching goal of this program is to support these children in optimizing their behaviors as a predictor of achieving positive developmental, behavioral, academic, physiological, and socioemotional outcomes in the future.^5^ Prevalent interventions implemented across these programs involve strategies and techniques in behavioral de-escalation, early intervention, acquisition of positive individualized coping skills, distraction to reduce self-injurious behaviors, pharmacologic and nonpharmacologic approaches in behavioral management.^5^

 As early intervention is a significant focus for these children, inpatient neurobehavioral units could provide the space for timely behavioral management which could further serve as a predictor of optimal treatment planning and care management in the future. Furthermore, this form of early intervention in this space could potentially mitigate modifiable risk factors inclusive of psychiatric comorbidities and in turn further mitigate more restrictive treatment options (e.g. residential and group home placements) in the future. In addition, the neurobehavioral unit could present a more controlled environment with resources on standby in establishing regimens for managing physiological symptomology that oftentimes are a part of developmental disabilities (e.g. constipation, dehydration, wet diapers generating discomfort). Given that these symptoms could contribute to behavioral escalation, focusing on them as targets for intervention could also be a predictor of optimizing future health and developmental outcomes for these children.

## Current state of inpatient pediatric neurobehavioral programs in the U.S.

 Given the missing link in our healthcare system to meet the needs of these children, this subgroup has grown as a neglected pediatric patient population. In fact, the demand for inpatient pediatric neurobehavioral programs exceeds the existing supply across the U.S. Currently in the U.S., there are 76 inpatient neurobehavioral programs for children. New Hampshire houses the highest number of these programs (15) followed by 10 programs in Illinois. The numbers across the rest of the states take a sharp declining turn: Pennsylvania houses five inpatient pediatric neurobehavioral programs followed by four programs each in Indiana and Iowa. Next there are three programs each in Massachusetts, Michigan, New Jersey, and Ohio.

 Five states (Colorado, Connecticut, Florida, Maryland, and Rhode Island) each house two inpatient pediatric neurobehavioral programs. The following sixteen states offer one inpatient pediatric neurobehavioral program: Alabama, California, Georgia, Kentucky, Maine, Minnesota, Mississippi, Missouri, Nebraska, New York, North Carolina, South Carolina, Tennessee, Texas, Virginia, and Wisconsin.

 Twenty-one states in the U.S. are without inpatient pediatric neurobehavioral programs. These states are the following: Alaska, Arizona, Arkansas, Delaware, Hawaii, Idaho, Kansas, Louisiana, Montana, Nevada, New Mexico, North Dakota, Oklahoma, Oregon, South Dakota, Utah, Vermont, Washington State, Washington DC, West Virginia, and Wyoming.

 Of note, not all inpatient pediatric neurobehavioral programs account for the range of developmental disabilities among children; for example, some do not provide interventions for intellectual disabilities or may only provide interventions for cerebral palsy. Table 1 presents a comprehensive breakdown and full listing of inpatient programs offered across states.

**Table 1:** Comprehensive breakdown of inpatient pediatric neurobehavioral programs in the United States

**State**	**Number of Programs **	**Names of Programs **
Alabama	1	Children’s of Alabama The Ireland Center Behavioral Health Inpatient Unit
Alaska	0	Not applicable
Arizona	0	Not applicable
Arkansas	0	Not applicable
California	1	UCLA Resnick Neuropsychiatric Hospital
Colorado	2	1) Children’s Hospital Colorado Neuropsychiatric Special Care Unit; 2) The Children's Hospital Neuropsychiatric Special Care Unit
Connecticut	2	1) Autism Inpatient Unit Hospital for Special Care; 2) Hospital for Special Care Autism Center
Delaware	0	Not applicable
Florida	2	1) Oppidan; 2) NeuroRestorative
Georgia	1	1) Inner Harbor Youth Villages
Hawaii	0	Not applicable
Idaho	0	Not applicable
Illinois	10	1) Streamwood Hospital; 2) Lurie CHC; 3) Northwestern; 4) Streamwood Behavioral Health; 5) Amita Health; 6) Advocate Children’s Hospital; 7) Linden Oaks; 8) Rogers Behavioral Health; 9) Riveredge Hospital; 10) Broadstep
Indiana	4	1) Harsha Behavioral Center; 2) NeuroDiagnostic Institute; 3) Wernle Youth & Family Treatment Center; 4) Damar
Iowa	4	1) League of Human Dignity; 2, 3, and 4) Optimae LifeServices (3 sites)
Kansas	0	Not applicable
Kentucky	1	River Valley Behavioral Health Hospital Behavior Intervention Program
Louisiana	0	Not applicable
Maine	1	Spring Harbor Hospital
Maryland	2	1) Kennedy Krieger Institute; 2) Sheppard Pratt
Massachusetts	3	1) Computational Behavioral Science Lab; 2 and 3) May Institute - Residential Services (2 sites)
Michigan	3	1) Harbor Oaks Hospital; 2) Pine Rest Christian Mental Health Services; 3) Stonecrest Behavioral Health Hospital
Minnesota	1	University of Minnesota Division of Child Psychiatry
Mississippi	1	Millcreek Of Magee Treatment Center
Missouri	1	St Louis Children’s Hospital
Montana	0	Not applicable
Nebraska	1	Broadstep
Nevada	0	Not applicable
New Hampshire	15	1) Hampstead Hospital; 2) Alternative Programs And Treatment; 3) Siddharth Services, Inc.; 4) Rose Meadow; 5) NeuroRestorative; 6) Visions for Creative Housing Solutions; 7) Toward Independent Living and Learning, Inc.; 8) The PLUS Company; 9) The Moore Center; 10) Next Steps Community Services; 11) North Country Independent Living; 12) Robin Hill Farm; 13) Community Partners for Change Inc. (CPCI); 14) The BrockHome; 15) Independent Services Network, Inc.
New Jersey	3	1 and 2) Bancroft Children's Residential Treatment Programs (2 sites); 3) Broadstep
New Mexico	0	Not applicable
New York	1	Upstate Cerebral Palsy
North Carolina	1	Broadstep
North Dakota	0	Not applicable
Ohio	3	1) Cincinnati Children’s Hospital Medical Center Division of Child and Adolescent Psychiatry; 2 and 3) I Am Boundless (2 sites)
Oklahoma	0	Not applicable
Oregon	0	Not applicable
Pennsylvania	5	1) Western Psychiatric Institute and Clinic of UPMC Center for Autism and Developmental Disorders; 2) Western Psychiatric Institute Western Pennsylvania Regional Center for Autism; 3) Foundations Behavioral Health; 4) Rogers Behavioral Health; 5) Devereux Pennsylvania Neurobehavioral Unit
Rhode Island	2	1) Bradley Hospital Center for Autism and Developmental Disabilities; 2) Butler Hospital Child and Adolescent Intensive Treatment Unit
South Carolina	1	Broadstep
South Dakota	0	Not applicable
Tennessee	1	Oak Plains Academy
Texas	1	Nexus Children's Hospital
Utah	0	Not applicable
Vermont	0	Not applicable
Virginia	1	Cumberland Hospital
Washington	0	Not applicable
Washington DC	0	Not applicable
West Virginia	0	Not applicable
Wisconsin	1	Broadstep
Wyoming	0	Not applicable

## Limitations in existing outpatient and school-based resources

 Waiting lists for outpatient services are also extensive. In addition, uninsured and underinsured patients may not be eligible for both inpatient and outpatient programs. In-home services are not meant to be continuous – these programs could be a bridge until a child is enrolled into an intensive day program or a specialized school.^3^ Of note, in-home programs can make the determination at any given time on whether a child can be discharged based on one of the following: (1) whether goals are met or (2) in instances when goals are unable to be achieved, whether the child’s developmental and behavioral needs require a higher level of care beyond the care that the program can provide.^3^ In addition, there are substantial regional variations in both intensive day programs and inpatient neurobehavioral programs for children across states in the U.S.

 The process of enrolling a child into a non-public school is not a simple process to navigate – this process takes substantial time. Furthermore, it is possible that non-public schools may have waiting lists. Also, placements that are identified may not always be accessible to families given transportation, childcare and other psychosocial determinants. During this time, the home school for the child in their jurisdiction could identify a bridge school within a public school setting that houses a special education program as part of securing free appropriate public education for the child.^6^ However, the reality is that the special education environments across public school settings will not always have the resources to meet the developmental and behavioral needs of the child as delineated in the individualized education plan, thereby potentially presenting a suboptimal therapeutic environment for the child.

 Developmental Disability Administration waivers, Autism waivers, model waivers, and other forms of waivers are in existence for a range of developmental disabilities and neurobehavioral disorders.^3,4^ Similar in context to identifying an appropriate school placement as well as inpatient and outpatient programs, the waiting lists for waivers could last from months to years. However when a waiver of this nature is active for a child, this waiver will ultimately waive any prior financial ineligibility and ensure that coverage for a range of resources and services are available to the child irrespective of the existing form of health insurance coverage for the child.^3,4^

## Suboptimal environmental conditions amidst awaiting treatment

 Of note, some inpatient pediatric neurobehavioral programs require a child to remain in a hospital leading up to admission, either in the form of boarding in the emergency department or admitted to a medical unit. Both the emergency department and medical unit present subtherapeutic environments for the child. Furthermore, both settings are not medically indicated for the child in this context and in turn are solely functioning as a placeholder requisite for admission to an inpatient neurobehavioral program. Moreover, the environment of a medical setting is not entirely safe for this vulnerable pediatric patient population - sharp containers are found in every room, there is significant fall risk from the furniture and windowsills where windows are present, continuous sitters are not available, and medical frontline staff are not trained in caring for these children. In fact, sitters may not be prioritized for self-harming behaviors unless patients are suicidal which can be challenging to assess with these patients since they are oftentimes non-verbal.

## Limited support for caregivers

 The truth is that this pediatric patient population is not well-supported by our existing healthcare system. Resources are not reaching children and their families fast enough. Furthermore, there are not enough resources to provide the foundation for behavioral stabilization as a precursor to support these children as they navigate the rest of their lives and optimize their quality of life across so many domains including activities of daily living and integration into society. In our own practice, we observe firsthand caregivers of these children expressing their desire to explore relinquishing custody or believing that their child needs to be hospitalized in a medical setting for self-injurious behaviors when the appropriate resources are not available. In each of these instances, caregivers are at their wit’s end and desperately searching for help. In their minds, bringing their child to the hospital is a mode of seeking help. These instances are a jarring reminder of the inequities inherent across our healthcare system in meeting the complex needs of this fragile patient population.

## Final thoughts

 Investment of time and resources is warranted in addressing this growing issue in the child and adolescent population with developmental disabilities and neurobehavioral disorders. Future work in this domain could contribute towards a more equitable and inclusive healthcare system and optimize a range of outcomes for these patients. It is crucial now than ever to support our children and adolescents in navigating the chronicity of their developmental disabilities and neurobehavioral disorders. As increasingly more people are living longer with disabilities, it is ever more imperative for existing healthcare systems to match population trends across communities on both national and global levels. Taking a health systems approach could yield a substantial downstream impact that not only aligns with patient and family preference and acceptability across a range of services and resources for this patient population but could also heighten cost efficiency across the healthcare system.

 There also needs to be further parity in approaching the constellation of these specialized conditions as chronic illnesses that warrant continued care and treatment similar in nature to lifelong medical conditions. Achieving this parity could also further contribute towards the larger goals of the World Health Organization, Healthy People 2030, and Sustainable Development Goals of the United Nations in delivering inclusive, equitable, and accessible healthcare services for individuals with disabilities.

## Author Contributions

 Conceptualization, writing (original draft preparation), and writing (review and editing) of the manuscript were completed by Aysha Jawed. Writing (review and editing) of the manuscript was completed by Christine Peck.

## Funding

 Not applicable.

## Availability of Data and Materials

 Not applicable.

## Ethical Approval

 Not applicable.

## Competing Interests

 The authors declare that they have no competing interests.
